# The American Journal of Insanity: Edited by the Officers of the New York State Lunatic Asylum

**Published:** 1844-10

**Authors:** 


Art. VI.—*The American Journal of Insanity:* edited by the Officers of the New York State Lunatic Asylum.
The care of the human mind is the most noble branch of medicine.—*Grotius.*
Utica: Bennett, Backus & Hawley. 1844.

We have received the first number of a journal of the above title, which is to be continued quarterly, and which we notice under the present head, rather than in our editorial department, because we propose extracting from it some highly interesting cases. The number before us has all the charm of a novel. We handed it to some unprofessional friends, to read the article on *Illustrations of Insanity*, and they declared that they were not able to lay it down until they had read the whole number. We have no doubt it is destined to a wide popularity, and, for ourselves, we shall look with impatience for the days of its publication. Our readers, we are sure, will thank us for the opportunity of reading the following cases:

*Sudden attack of Insanity, and instantaneous recovery.*—Mr. ——, aged 48, had uniformily enjoyed good health until the summer of 1842, when he complained some of not feeling well, was weak and dyspeptic, and in November had what was supposed to be a paralytic attack. For this and severe pain of the head he was bled *seven* or *eight* times, took cathartic medicines and was blistered largely. He remained dull and disinclined to exercise for five or six weeks, when he became suddenly deranged. The immediate cause of his derangement, was the entrance of a sheriff to take his property for debt.

Early in March, 1843, he was admitted into the Asylum. He appears idiotic, timid, thinks robbers are pursuing him; is inoffensive, and readily submits to whatever is requested, with the exception of being shaved, because, he says, 'It will take away his strength, and he cannot consent to it until after the war is over.'

The second day after his arrival, he was told in a decided manner by the physician that the war was ended. 'Is it,' says he, 'what has General Jackson done with those rascals, hung them?' Answer, yes. 'Hurrah, hurrah,' he exclaimed, 'that is right, I will now be shaved;' and readily and pleasantly submitted to have his beard of some six weeks' growth removed.

He had a warm bath, and as he was feeble and pale, he was put on an invigorating diet and the use of tonics. He took large doses of the precipitated carbonate of iron, combined with the extract of conium, three times a day, and his general health and appearance began to improve. His appetite became good, and he sleeps well. During the day, he amused himself by talking and laughing with other patients, and in playing cards and other games.

A few weeks after this he was invited into the office of the Superintendent, with whom he conversed some time in his usual disconnected manner, as if he did not know what he was saying, when looking around the room, he asked, 'was I ever in this room before?' He was told he was when he first came. He then asked, 'what town is this?' Answer, Utica. After reflecting a short time, he remarked, 'then I am in the Lunatic Asylum I know.' From that moment his mental powers were restored.

Instead of returning the same evening to the apartment he had occupied, he was placed in a different story of the building, and in the morning, when he was informed that he had heretofore occupied another, he was anxious to visit it, but on returning to it, he had no recollection of ever having been there before, and although he recollected his associates, he had not the least remembrance of anything he had said or done since he had been at the Asylum, until the evening alluded to.

The last thing he recollected was the entrance of the sheriff, as we have mentioned. He was discharged well, and still enjoys good health.

Was not the delirium in this case, produced by the extensive loss of blood? Cases somewhat analogous, and which may serve to elucidate this, may be found in Marshall Hall's Researches relative to the morbid and curative effects of loss of blood.

*Duration of insanity three years—complete recovery.*—Mr. ——, aged 55, large frame, large well-formed head, with vigorous intellect highly cultivated, experienced a slight paralytic attack when a young man, from which, however, he soon recovered. He also had one or two short attacks of insanity previous to the present one, which was caused apparently by too great and constant mental exertion and political excitement.

He is usually quite harmless, and sociable, imagines himself the Prince of Wales and Emperor of the world. His bodily health seems tolerably good, though at times he is dyspeptic and bilious, which a few blue pills and laxatives remove. In this state he continued with but little variation nearly three years, busily engaged in reading, writing, and conversing, and mostly for the purpose of establishing or asserting and making known throughout the world his right to govern it. For this purpose he addressed numerous letters to the rulers of different countries. The following is a specimen:

"Mahomet Ali, Governor
of Egypt.

"Dear Sir:

"I was born in the city of Philadelphia, July 26, 1789. On the father's side am descended from the Roman Emperor Constantine who built Constantinople in the 4th century. On the mother's side I am descended from Mary Stuart, daughter of James V., King of Scotland. It is my intention to dethrone the Sultan if I live long enough—none of the crowned heads in Europe have any right to reign, and will be one and all dethroned either by me or my successors. A son of Joseph Bonaparte, ex-King of Spain, who is my nephew, is the intended King of Italy. The Pope and all other divines will be taught to mind their appropriate business, the cure of souls. I can and will get along without them. I regret the destruction which was made among my subjects at Acre. We shall have a war with England which will end in the overthrow of the tyrannical House of Hanover. I can in one campaign take every inch of territory which that house possesses on this continent. I have been confined myself for nearly two years and prevented from supporting you in your contest with the Allied Powers. The downfall of the Thiers ministry in France, prevented the French from aiding you. Thiers is my friend, so is Mr. O'Connell, and so is every Republican in Europe, and some of the Nobility. I care very little for the Nobility. William the Conqueror was a bastard and never conquered the Britons. My ancestors sought an asylum in these western wilds, and have not yet been conquered. I have some of the native Indian blood in me of the Mohawk Tribe, and mean to teach our white oppressors that we owned this country before the discovery of Columbus. The race of men are bound to obey me as their lawful head. I intend to conquer my inheritance, and then see if I can't govern it better than it has been. I was placed under the protection of General Washington, and have married his grand-daughter. It is my intention to tread in the footsteps of that great man. His limits were the United States. My government embraces the world, and of course must be a military one. The world always has, after a fashion, been governed by the sword, but there has been too many commanders-in-chief. The world will be at peace only so fast as it obeys me.

"I am, very respectfully,

Yours, &c.,

"——— ———, Prince

"of Wales, and Emperor of the world.

"P. S. The Emperor Napoleon was my uncle, having married a sister of my mother for his first wife."

He was always gentlemanly in his conduct, and in his conversation on topics not relating to himself, interesting and instructive.

Various plans were adopted to withdraw his mind from his particular delusions, but without effect. At one time political and historical works were withheld from him, and he was furnished with works on natural history. For awhile he talked less about his supreme command of men, but formed projects for improving the races of other animals, and was for sending agents and directions throughout the world for the purpose.

His time was not, however, misspent. He read much and systematically, taking notes frequently, and in this way his mind if not improved (it probably was) did not become weakened.

His recovery seemed not to arise from any particular treatment, though an attack of bronchitis preceded the change and improvement of his mind.

The case we deem an encouraging one, and should lead us not to despair even if no improvement is observed for two or three years in similar cases. Generally we consider cases of insanity that have uninterruptedly continued three or even two years, as probably incurable, but in some cases even of much longer continuance recovery takes place. So long as there is any hope, great pains should be taken to cultivate the mental powers, and to keep them active. Hence schools are of service in Lunatic Asylums. Y.

